# Whipple Procedure With Preservation of the Right Gastroepiploic Artery After an Ivor Lewis Esophagectomy

**DOI:** 10.7759/cureus.103756

**Published:** 2026-02-17

**Authors:** Ergin Erginöz, Dogus C Ekdal, Ender Dulundu

**Affiliations:** 1 Surgery, Memorial Sloan Kettering Cancer Center, New York, USA; 2 General Surgery, Marmara University, Istanbul, TUR; 3 General Surgery, Istanbul University-Cerrahpaşa, Istanbul, TUR

**Keywords:** gastroduodenal artery, ivor lewis esophagectomy, pancreaticoduodenectomy, right gastroepiploic artery, whipple procedure

## Abstract

In patients with a history of Ivor Lewis procedure for esophageal cancer, the majority of the arteries that supply the stomach are ligated. Therefore, the blood supply of the stomach becomes dependent on the gastroduodenal artery. The arterial supply of the stomach could be compromised if a patient undergoes another surgical procedure, such as a Whipple procedure, that involves ligation of the gastroduodenal artery.

A 73-year-old woman with a history of an Ivor Lewis procedure for esophageal adenocarcinoma presented to the clinic with symptoms of abdominal pain, vomiting, and jaundice. Upon workup, the CT scan revealed a lesion in the pancreatic head. She underwent a Whipple procedure with the preservation of the gastroduodenal artery, and therefore, the right gastroepiploic artery.

The blood supply to the gastric pull-up reconstruction following esophagectomy is dependent mostly on the right gastroepiploic artery. During the Whipple procedure, the gastroduodenal artery is cut and ligated. This would not be suitable for patients with a history of esophagectomy, as it would compromise the blood supply to the stomach.

Pancreaticoduodenectomy can be successfully performed in patients who have had an Ivor Lewis esophagectomy with the gastroduodenal and right gastroepiploic arteries preserved.

## Introduction

Pancreatoduodenectomy, also known as the Whipple procedure, is a complex surgical technique that involves the removal of the pancreatic head, duodenum, common bile duct, and, sometimes, the pylorus region. It is indicated for cancers located at the head of the pancreas, pancreatic neuroendocrine tumors, gastrointestinal stromal tumors, intraductal papillary mucinous neoplasms, duodenal adenocarcinomas, and adenocarcinoma of the ampulla of Vater [1-5]. In some cases, it is performed in severe pancreatic trauma and in cases where an inflammatory mass forms in the head of the pancreas due to chronic pancreatitis [1,6,7].

In the Whipple procedure, the gastroduodenal artery (GDA) is cut and ligated at its origin so that there is a loss of blood supply to the right gastroepiploic artery (RGEA). Under normal conditions, the loss of blood supply to the RGEA does not compromise the arterial blood supply to the stomach since there are a vast number of anastomoses that supply the organ. However, in patients who have altered anatomy due to a previous surgery, such as the Ivor Lewis procedure, most of the arteries that supply the stomach are ligated. Therefore, the blood flow through the RGEA becomes essential for the arterial supply of the greater curvature of the stomach [8]. In this report, we present the case of a 73-year-old woman who had a history of Ivor Lewis procedure and was diagnosed with adenocarcinoma of the pancreatic head, where she underwent Whipple procedure with preservation of the RGEA. The aim of this report was to describe the surgical planning and technical feasibility of performing pancreaticoduodenectomy in a patient with prior Ivor Lewis esophagectomy, with particular emphasis on preservation of the gastroduodenal and right gastroepiploic arteries to maintain adequate perfusion of the gastric conduit.

## Case presentation

**Figure 1:**
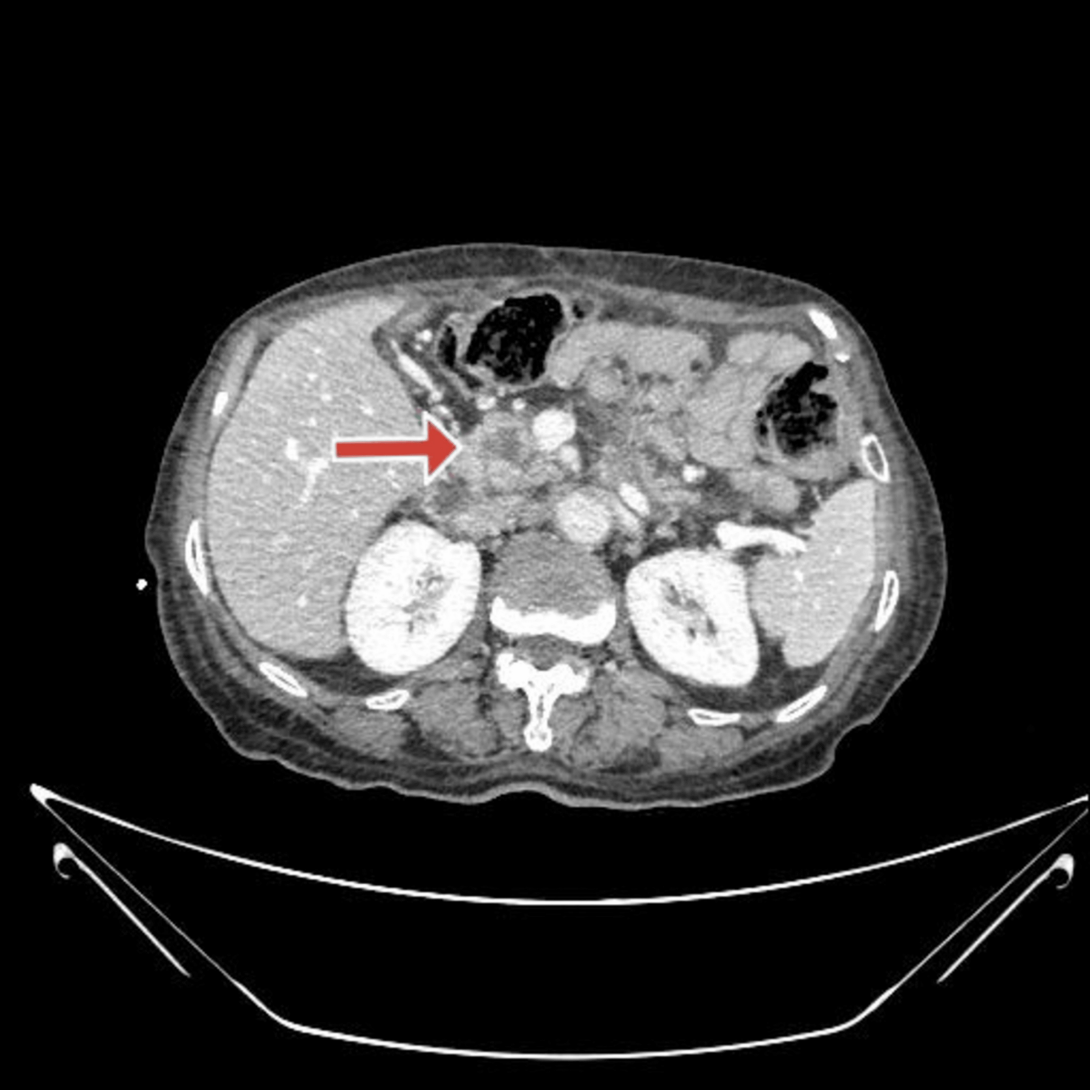
The CT scan of the patient showing a 2 cm lesion in the pancreatic head.

A 73-year-old woman presented with symptoms of abdominal pain, nausea, vomiting, and jaundice. Five years ago, the patient was diagnosed with esophageal cancer for which she underwent Ivor Lewis esophagectomy, and the pathological result was consistent with esophageal adenocarcinoma (T2N0M0, with negative surgical margins). Past medical history was unremarkable. She was operated on for a lumbar hernia 5 years ago and a tonsillectomy 10 years ago. The physical examination was normal except for jaundice upon inspection. Laboratory values were normal except for hemoglobin 10.6 g/dL (reference: 13.6-17.2), platelet count 129.000 μL (reference: 156.000-373.000), glucose 149 mg/dL (reference: 74-109), total bilirubin 2.56 mg/dL (reference: 0.2-1.2), direct bilirubin 1.17 mg/dL (reference: <0.3), and CA 19-9 36.9 U/mL (reference: <35). Upon workup, the contrast-enhanced computed tomography (CT) revealed a 2 cm hypodense lesion in the pancreatic head and a dilated common bile duct with a diameter of 18 mm (Figure 1).

The positron emission tomography (PET) scan revealed a 2 cm lesion in the pancreatic head with increased uptake of F-fluorodeoxyglucose (FDG). There were also enlarged lymph nodes within the left para-aortic region, interaortocaval region, and peripancreatic region. Although FDG-avid lymph nodes were detected on PET imaging, no distant organ metastasis was identified on contrast-enhanced CT and PET-CT. Intraoperative exploration further confirmed the absence of peritoneal or hepatic metastases, and the mass was deemed suitable for curative resection.

Preoperative surgical planning included careful evaluation of the vascular anatomy on contrast-enhanced CT imaging, with specific attention to the patency and course of the GDA and RGEA to ensure preservation of the gastric conduit blood supply during pancreaticoduodenectomy. Although 3D-CT angiography was not performed, intraoperative assessment confirmed the feasibility of preserving these vessels to maintain gastric conduit perfusion.

**Figure 2:**
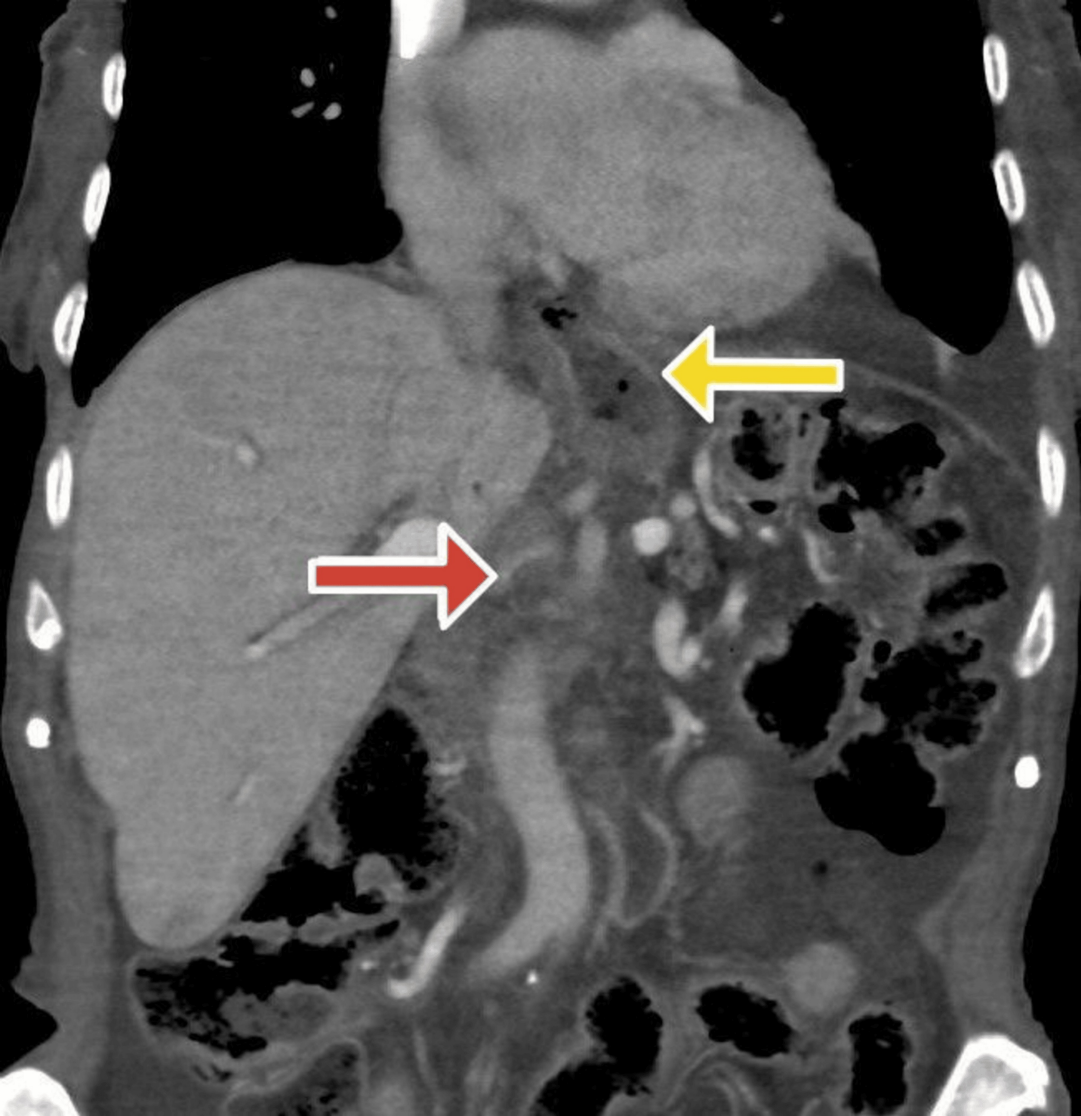
The CT scan displaying the preservation of the gastroduodenal artery (red arrow) with contrast enhancement of the stomach wall (yellow arrow), suggesting arterial supply from the RGEA.

We used a midline incision for this operation. Upon inspection, a mobile mass was palpated within the pancreatic head, and there were no other gross metastatic lesions in other organs. The gastrocolic ligament was opened, which allowed access to the lesser sac. The hepatoduodenal ligament was opened, and careful dissection was performed. The transverse colon was mobilized, and the duodenum was dissected from its retroperitoneal attachments. A Kocher maneuver was performed to mobilize the duodenum from its lateral attachments. Dissection was carried out toward where the superior mesenteric vein crossed the third portion of the duodenum. The third and fourth portions of the duodenum were mobilized. The common bile duct and GDA were carefully dissected, and vessel loops were placed around them. Usually, the GDA is ligated from its origin, the proper hepatic artery. However, in this case, given the patient’s Ivor Lewis operation history, the GDA and RGEA were spared to supply the gastric tube (Figure 2).

**Figure 3:**
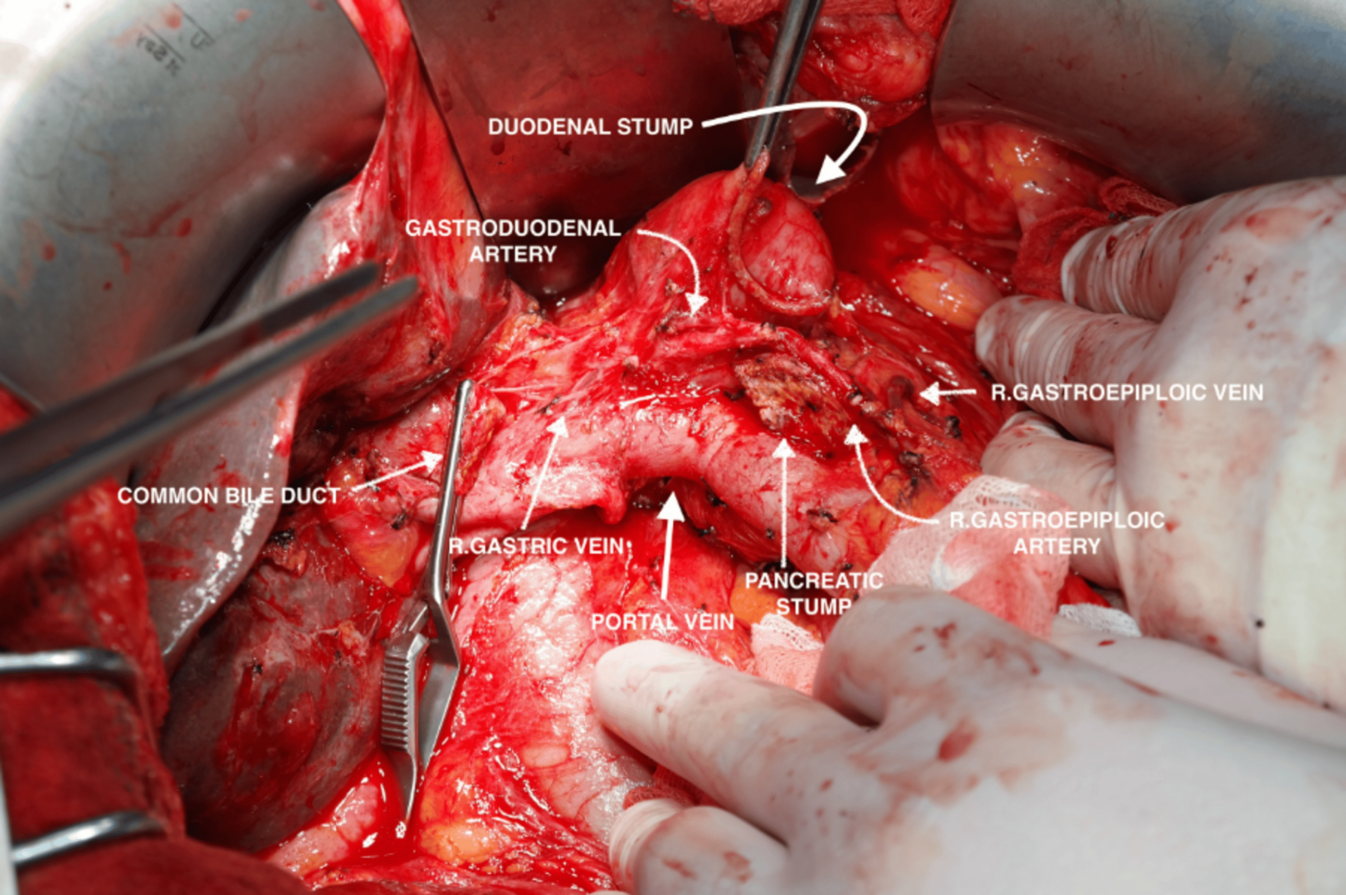
Intraoperative location of the gastroduodenal artery with respect to surrounding anatomical structures following the Whipple procedure

The pancreas and the proximal duodenum together were transected just distal to the pylorus as part of a pylorus-preserving pancreaticoduodenectomy. A feeding catheter was inserted into the pancreatic duct. The procedure was completed with hepaticojejunostomy, pancreaticojejunostomy, and antecolic duodenojejunostomy anastomoses (Figure 3).

In the postoperative period, the patient developed cardiac arrhythmia requiring close monitoring and medical management, which contributed to a prolonged hospital stay of 37 days. No major surgical complications such as pancreatic fistula, anastomotic leak, or postoperative hemorrhage were observed. Recovery was further supported with gradual nutritional advancement and multidisciplinary care. The patient was discharged after 37 days. The pathological result was consistent with G3 poorly differentiated ductal adenocarcinoma of the pancreas (T2N2). She did not receive any neoadjuvant treatment. As adjuvant therapy, the patient was treated with capecitabine, gemcitabine, 5-fluorouracil, oxaliplatin, and irinotecan. After six months of follow-up, the patient was symptom-free. The patient was lost to follow-up one year after surgery.

Written informed consent was obtained from the patient.

## Discussion

Cancer of the pancreas has a high morbidity and mortality, with a global incidence of 5.5 per 100,000 for men and 4.0 per 100,000 for women [9]. It occurs more commonly in men, and it is predominantly a disease of the older population, where most patients are aged more than 50 years of age [10,11]. Although various modifiable and non-modifiable risk factors have been identified, pancreatic cancer can develop as a primary tumor following the diagnosis of another tumor. This patient had a history of esophageal adenocarcinoma, for which she was treated five years ago.

Development of a second primary malignancy after esophageal cancer is reported to have an incidence between 8.3% and 27.1% [12]. The location of a second primary malignancy following esophageal cancer seems to vary between different countries. The secondary malignancy tends to develop within the stomach, head, and neck in Japan, China, and Europe [12,13]. In the United States, the location of the secondary malignancy after esophageal adenocarcinoma mostly involved the pancreas, oropharynx, kidney, and renal pelvis, while that of esophageal squamous cell carcinoma involved the oropharynx, thyroid, lung, and bronchus [12]. The most important risk factor for the development of esophageal cancer and the second primary tumor is smoking [12,13]. Amin et al. have looked at the association and incidence of pancreatic adenocarcinoma in patients with a history of non-pancreatic primary tumors [14]. They have found that, among patients between the ages of 20 and 49 years, the risk of developing pancreatic cancer increased in patients with a primary tumor involving the ascending colon, hepatic flexure, biliary system, uterine cervix, testes, breast, and hematopoietic system [14]. Between the ages of 50 and 64 years, the risk of developing pancreatic cancer increases after cancers of the stomach, hepatic flexure, lung, bronchus, pharynx, and bladder [14]. After 65 years, the risk increased after cancers of the stomach, hepatic flexure, biliary system, and uterus [14]. Overall, the higher risk of developing pancreatic cancer following a primary tumor was associated with tobacco-associated malignancies, various genetic syndromes (familial adenomatous polyposis, hereditary non-polyposis colorectal cancer, Peutz-Jeghers syndrome, *BRCA 1/2* mutations, and familial atypical multiple mole melanoma syndrome), cancers that are treated with radiation, and environmental factors such as *Helicobacter pylori* infection [13,14].

The blood supply to the gastric pull-up reconstruction following esophagectomy is accomplished mostly on the RGEA, which is a branch of the GDA. Similarly, venous drainage occurs through the right gastroepiploic vein (RGEV). During the Whipple procedure, the GDA is typically cut and ligated. This would not be suitable for patients with a history of esophagectomy, as it would compromise the blood supply to the stomach. According to Inoue et al., three different techniques can be used to establish the continuation of the blood supply to the stomach: (1) pancreaticoduodenectomy with preservation of the GDA, RGEA, and RGEV; (2) pancreaticoduodenectomy with divisions of the GDA, RGEA, and RGEV, following microvascular reconstruction; (3) pancreaticoduodenectomy with divisions of the GDA, RGEA, and RGEV, removal of the gastric conduit, and reconstruction of the gastrointestinal tract using the small or large intestine [15].

Different techniques have been preferred by various surgeons in patients with a history of gastric tube reconstruction for esophageal cancer. Izumi et al. performed subtotal stomach-preserving pancreaticoduodenectomy without resecting GDA, RGEA, RGEV, and the right gastric artery [16] Fraguilidis et al. preserved RGEA, Inoue et al. preserved RGEA and RGEV, Okochi et al. preserved RGEA, and Orii et al. preserved RGEA, RGEV, right gastric artery, and right gastric vein [15,17-19]. In our case, we have preserved the GDA.

When planning a Whipple procedure following esophagectomy, it is important to confirm the positioning of the vessels before surgery with a 3D CT angiography [16]. Verifying the positioning and blood flow to the gastric tube and the pancreas may allow for the preservation of the gastric tube blood supply without resecting GDA and RGEA.

## Conclusions

Whipple procedure can be safely performed in patients with a history of Ivor Lewis esophagectomy with preservation of the GDA and RGEA.
